# Carbon regime structures functional trait trajectories during primary succession in microorganisms

**DOI:** 10.1093/ismejo/wrag134

**Published:** 2026-05-23

**Authors:** Grace A Cagle, Benjamin Baiser, Jessica R Bernardin, Leonora S Bittleston, Erica B Young, Sarah M Gray, Zachary B Freedman

**Affiliations:** Department of Soil and Environmental Sciences, University of Wisconsin-Madison, Madison, WI 53706, United States; Department of Wildlife Ecology and Conservation, University of Florida, Gainesville, Florida 32603, United States; Department of Biological Sciences, Boise State University, Boise, ID 83725, United States; Department of Biological Sciences, Boise State University, Boise, ID 83725, United States; Department of Biological Sciences and School of Freshwater Sciences, University of Wisconsin-Milwaukee, Milwaukee, WI 53204, United States; Department of Biology-Ecology and Evolution, University of Fribourg, Fribourg, CH-1700, Switzerland; Department of Soil and Environmental Sciences, University of Wisconsin-Madison, Madison, WI 53706, United States

**Keywords:** primary succession, rRNA copy number, genome size, life-history strategy, bacterial colonization, traits

## Abstract

Primary succession is a foundational process in ecology, but how microbial communities shift functionally during succession, and whether these dynamics follow predictable patterns, remains unresolved. We conducted a systematic review of functional primary succession in microorganisms and applied a consistent metagenomic pipeline to evaluate functional richness, rRNA operon copy number (RRN), and average genome size (AGS) over time. We also explored the yield–acquisition–stress life-history framework using functional gene annotations. Across autotrophic systems, RRN tended to decrease and AGS tended to increase during succession, whereas heterotrophic systems exhibited more variable trajectories. These consistent shifts in autotrophic systems suggest a transition from early colonization by copiotrophic taxa with small genomes and high RRN toward later-stage communities with larger genomes, lower RRN, and greater functional versatility. In contrast, heterotrophic systems showed heterogeneous trait trajectories, likely reflecting variation in the timing and predictability of organic inputs. Topic modeling further revealed that early successional stages were enriched in stress-tolerance genes, followed by shifts toward other strategies over time. While certain trait patterns such as RRN and AGS appeared broadly conserved, changes in life-history strategies during succession were context dependent and shaped by resource dynamics and system type. These findings suggest that microbial successional trajectories are structured by differences in resource availability, particularly whether systems are driven by autotrophic inputs or constrained by externally supplied carbon sources.

## Introduction

Succession describes directional changes in species populations, community structure, and ecosystem functioning over time, typically following the creation of new substrates or the removal of established biota [1, 2]. Although succession theory is well developed in plant and animal communities, its application to microorganisms, defined here as prokaryotes, remains comparatively underexplored, despite increasing evidence that microbial communities also exhibit directional functional and compositional change during succession [3, 4]. Evidence from soils suggests that microbial communities may transition from fast-growing, resource-acquisitive taxa (*r*-strategists) to slower growing taxa with greater resource-use efficiency (*K*-strategists) [4–8]. These observations suggest that microbial primary succession may involve predictable trade-offs in life-history strategy, yet few studies have evaluated whether these dynamics are consistent across diverse successional environments, including newly exposed soils and rock surfaces, host-associated tissues and engineered environments [9]. However, most studies of microbial succession have emphasized taxonomic turnover, even though shifts in taxonomic composition do not necessarily capture changes in ecological strategy or community function.

Functional traits offer a complementary and mechanistic framework by linking community composition to growth strategies, resource use, and ecosystem processes [10]. Focusing on trait dynamics during primary succession therefore enables explicit tests of whether microbial communities follow consistent functional trajectories, and whether those trajectories vary across systems characterized by different resource constraints. Such analyses can be grounded in life-history strategies that capture fundamental trade-offs in growth, resource use, and stress tolerance [11]. While categorical life-history assignments applied broadly across taxa have limited predictive power in microbial systems, microbial strategies can be represented as continuous trait distributions that vary with ecological context [12]. Accordingly, in microorganisms these life history strategies have been inferred primarily through genomic traits rather than taxonomic classification [13–16].

Two traits especially relevant for studying microbial succession across systems are rRNA operon copy number (RRN) and average genome size (AGS), which consistently differentiate taxa along axes of growth rate and flexibility in resource use, respectively [17–19]. High RRN is generally associated with faster maximum growth rates and r-selected or copiotrophic tendencies, often at the expense of growth efficiency, whereas lower RRN is linked to slower but more efficient growth under resource limitation aligning with *K*-selected or oligotrophic strategies [20, 21]. AGS provides an orthogonal axis of strategy: larger genomes tend to include encoding for more complex metabolic and functional capabilities, enabling greater versatility in fluctuating or resource-limited environments [22, 23]. Although RRN and AGS can be weakly correlated across some taxa, they are not redundant and reflect distinct trade-offs in microbial ecology [18, 24]. The recurrence of RRN and AGS as predictors of microbial strategy motivates their use as interpretable indicators of functional change during primary succession, while allowing for context dependence across systems [24, 25].

Stress tolerance—specifically tolerance to resource-poor, variable or physically extreme conditions—is another major axis of microbial life-history variation that is especially relevant during early primary succession [26, 27]. Building on established trade-offs, the yield–acquisition–stress (Y–A–S) framework adapted Grime’s CSR model to microorganisms by conceptualizing strategies along three continuous trait axes reflecting growth efficiency, resource acquisition, and stress tolerance [28–30]. Here, the “Y” trait axis represents the amount, or yield, of microbial biomass produced per unit of resource consumed, which is maximized when resource limitation and stress are minimal. The “A” trait axis reflects that level of microbial investment in resource acquisition such as extracellular enzyme production and subsequent uptake of depolymerized substrates. Lastly, the “S” trait axis represents traits related to strategies to maintain cellular integrity and osmotic balance when a microbe is exposed to stress in the form of environmental conditions far outside their optimum range. The Y–A–S framework provides a mechanistic structure for interpreting how microbes balance energy efficiency, nutrient uptake, and stress tolerance, and has been applied to link microbial traits to ecosystem processes such as decomposition [31]. However, its application across systems remains limited by the need to determine which functional genes best represent traits related to Y, A, or S in any given environment [32]. Although genomic ordinations suggest that microbial strategies cluster along three principal axes [19, 33, 34], it is unresolved if these axes consistently structure trait dynamics during succession across systems.

In microbial systems, the source of carbon inputs is a primary determinant of successional trajectories across habitats colonized during primary succession, including host-associated, aquatic, and mineral substrata. Fierer *et al.* [9] formalized these patterns into a framework distinguishing autotrophic, endogenous heterotrophic, and exogenous heterotrophic succession based on carbon acquisition. Autotrophic systems begin with minimal organic carbon and generate it de novo via photosynthesis or chemolithoautotrophy. Endogenous heterotrophic systems rely on a finite, locally available organic carbon pool that is progressively modified by microbial activity. Exogenous heterotrophic systems receive continual external carbon inputs that microbes do not directly control. Whether functional trait dynamics consistently differ among these resource-defined successional patterns, however, is unknown.

Here, we use comparative metagenomics to evaluate if microbial primary succession is characterized by consistent shifts in functional traits across ecosystems and whether these trajectories differ by succession type. We combined a systematic review with standardized re-analysis of published metagenomes to assess changes in functional richness, RRN, AGS, and trait groupings inferred from gene annotations. By comparing autotrophic and heterotrophic systems, we tested whether ecological context structures successional patterns in microbial life-history strategies.

## Materials and methods

### Systematic literature review and study selection

We identified candidate studies of microbial primary succession with publicly available metagenomic data using a systematic literature review conducted in Web of Science (04/04/2024) following PRISMA guidelines (Supplementary Fig. S1; Supplementary Table S1; [35]). Search terms were developed targeting three required elements: bacteria, primary succession, and metagenomic sequencing (Supplementary material; Supplementary Table S2). Records were screened against the following criteria: (i) The study included ≥3 time points such that the earliest sampling point plausibly represented initial colonization of the substrate prior to substantial biological modification; (ii) sequencing chemistry generated reads suitable for functional annotation (≥100 bp); (iii) sequence data were available; (iv) at least one treatment captured primary succession without experimental manipulation of the colonizing community (e.g. exclusion of studies using curated inoculant or intentionally filtered microbial assemblages).

We further selected eight studies identified by our systematic review for re-analysis based on the following additional criteria: (i) The data were available through the sequence read archive (SRA) or European nucleotide archive (ENA) databases. This assured consistency in sequence format and metadata that allowed comparisons to be made among the studies using a consistent workflow. (ii) The study design included time-point replication. (iii) The study system was not the mammalian gut microbiome. Development of the mammalian gut microbiome has been well characterized, i.e. [36, 37], and represented 76% of all studies identified by our systematic review (54 of 71; see *Results*).

### Classification of successional systems

Studies meeting these criteria were then assigned as autotrophic or heterotrophic primary succession following the conceptual distinctions outlined by Fierer *et al.* [9]. Briefly, autotrophic succession encompassed systems in which primary colonization was expected to rely predominantly on in situ, autochthonous primary production or chemoautotrophy, whereas heterotrophic succession included systems dependent primarily on externally supplied, allochthonous organic carbon. These assignments (Table 1) were made based on ecological context and resource characteristics described in the original publications; no assumptions were made about the taxonomy or trophic identities of the earliest colonists. Although oral biofilms are host-associated in vivo, the oral biofilm system analyzed here was treated separately from host-associated microbiomes because succession occurred in a sterile microcosm under controlled conditions, without host immune regulation or tissue feedback. For glacial forefield systems, distance from the glacier terminus was used as a proxy for time since substrate exposure, consistent with the original study design.

**Table 1:** Ten study systems from eight published metagenomic studies of primary succession of microorganisms were identified from a systematic literature review and re-analyzed in this study.

Microbial primary succession system	Succession type	Description	Duration	N	Mean sequences per sample (million) ± s.d.	Data ID and citation
Volcanic eruption	Autotrophic	Examined plots near Llaima volcano affected by lava deposition in 1640, 1751, and 1957.	373 years	9	17.5 ± 1.5	PRJNA602600 [94]
Midtre Lovénbreen glacier forefield	Autotrophic	Assessed newly exposed terrain following glacier retreat; distance from the glacier edge was used as a proxy for time since exposure.	2000 ± 1000 years^†^^,^^a^ (1,650 km)	21	27.9 ± 16.2	PRJEB41174 [53]
Greenland Ice Sheet glacier forefield (GrIS)	Autotrophic	Assessed newly exposed terrain following glacier retreat; distance from the glacier edge was used as a proxy for time since exposure.	7000 ± 500 years^†^^,^^b^ (10,000 km)	20	71.7 ± 15.8	PRJEB41174 [53]
Storglaciären glacier forefield	Autotrophic	Assessed newly exposed terrain following glacier retreat; distance from the glacier edge was used as a proxy for time since exposure.	100 ± 50 years^†^^,^^c^ (370 km)	18	66.0 ± 10.9	PRJEB41174 [53]
Concrete seawall	Autotrophic	Evaluated biofilm formation on different stone materials used for sea wall construction in outdoor aquaria with natural seawater.	4 weeks	72	5.93 ± 0.7	PRJNA698754 [95]
Water pipe	Heterotrophic exogenous	Characterized biofilm development on a steel pipe in a grey water distribution system.	10 months	17	82.6 ± 6.5	PRJNA872219 [80]
Oral biofilm	Heterotrophic exogenous	Biofilms were grown in anoxic microcosm using human saliva samples as inoculating seeds.	10 days	75	79.3 ± 10.2	PRJNA983519 [96]
Biofilm reactor	Heterotrophic exogenous	Followed the start-up of a full-scale partial nitration-anammox moving bed biofilm reactor.	175 days	45	74.8 ± 19.3	PRJEB58181 [54]
Bumble bee gut	Heterotrophic exogenous	Characterized the gut microbiomes of bumble bee (*Bombus impatiens*) workers from three commercially reared colonies over their lifespan.	75 days	46	44.8 ± 4.5	PRJNA849590 [77]
Infant nasal passage	Heterotrophic exogenous	Characterized the development of the early infant nasal microbiome during the first year of life.	9 months	184	2.1 ± 5.8	PRJNA610982 [74]

^†^Distance from glacier front was reported. Temporal estimates are provided here for comparison with other studies.

a Estimate derived from [97].

b Estimate derived from [98].

c Estimate derived from [99].

### Metagenomic data processing and functional annotation

The re-analysis was conducted by the following steps. The raw sequences from each selected study were downloaded by project identifier from SRA and ENA using anvi’o [38]. To avoid assembly bias among studies with variable levels of coverage, we applied a short read-based approach. Briefly, protein coding regions were predicted from quality filtered reads using FragGeneScanRs [39]. The abundances of functional gene orthologs were determined by searching proteins against the KOfam database (KEGG; version 2019–09) using Hidden Markov Models (HMMs) with hmmer [40, 41]. KEGG was selected due to its functional focus and inclusion of HMMs for more accurate gene annotations for short reads. Genes were filtered to retain only KEGG orthologs (KOs) found in prokaryotes using the *keggrest* package in R [42]. Normalized relative abundances of genes were calculated in each sample based on the total number of reads. AGS and RRN were estimated as described by Pereira-Flores *et al.* [43]. Functional gene richness was calculated as the total number of unique KOs detected in each metagenome, reflecting the breadth of functional potential rather than total gene abundance. A detailed description of the bioinformatics workflow, along with information on samples excluded due to quality concerns or insufficient replication, is provided in the Supplementary material.

### Statistical analysis of successional trait trajectories

Temporal trajectories in gene richness, AGS, and RRN during succession were evaluated by fitting third-degree polynomials independently for each system. Because the number and spacing of time points varied among systems, we used system-specific polynomial regressions to visualize potential non-linear trajectories without imposing a shared functional form across studies. Cubic models were selected because they offer a low parameter but flexible option capable of representing monotonic shifts, unimodal patterns, and inflection points without imposing a mechanistic form. The best fitting curve for statistically significant models was selected based on the Bayesian Information Criterion (BIC) and a Chi square test between the regression models [44]. As a sensitivity analysis, we additionally fit generalized additive mixed models (GAMMs) for each trait separately, pooling systems within autotrophic and heterotrophic succession types. Here, “system” refers to a distinct successional trajectory, including individual glacier forefields treated separately throughout the analysis. In these models, time was modeled as a smooth term and system was included as a random intercept. These analyses were used to assess whether inferred temporal trends were robust to model specification rather than to estimate system-specific trajectories.

### Mixed-effects analysis of AGS–RRN relationships

To evaluate whether traits associated with microbial growth rate and genomic versatility covaried across samples, we fitted linear mixed-effects models testing the relationship between AGS and RRN using the lme4 package in R [45]. Models were fit separately for autotrophic and heterotrophic systems, with RRN as the response variable and AGS as a fixed effect. System identity was included as a random intercept to account for non-independence among samples within the same successional sequence. Models were fit using restricted maximum likelihood, and degrees of freedom and *P*-values for fixed effects were estimated using Satterthwaite’s approximation in the *lmerTest* package [46].

### Latent Dirichlet allocation of functional gene groups

To identify groups of genes that covary during succession, we applied Latent Dirichlet Allocation (LDA) [47], an unsupervised topic modeling approach (Supplementary Fig. S2). In this framework, samples were treated as “documents” and KOs as “terms”, following the standard analogy used in topic models. Here, “topics” represent groups of KOs identified by the model. For the LDA-based analysis, we restricted the input gene set to KOs present in the microTrait database [30] to facilitate downstream functional interpretation of the inferred gene groups. For these KOs, we calculated term frequency-inverse document frequency (TF-IDF) scores and retained genes in the top 20% within each study to reduce noise while retaining informative variation [48]. The resulting sample-by-gene matrices of relative abundances were used as input to LDA, with the number of topics fixed at three to reflect the hypothesized trade-offs among growth yield, resource acquisition, and stress tolerance strategies. Models were implemented using the *topicmodels* package in R [49] and run separately for each study. Outputs included θ (topic mixture weights per sample), representing the relative contribution of each inferred gene group to each sample, and β (gene weights per topic), representing the relative contribution of each KO to each inferred gene group. We used temporal changes in θ values to characterize how the relative weighting of gene groups shifted across successional time. The top 10 genes in each cluster were annotated using microTrait life-history strategy assignments, supplemented by manual classification for KOs lacking annotations.

The number of LDA groups contributing meaningfully to each system was evaluated using the *ldacov* package in R (Supplementary material; [50]). To assess how many inferred groups captured major patterns in gene composition, we calculated the average group proportions (θ) for each group across samples and determined the cumulative contribution of gene abundance represented as additional groups were included. These cumulative θ values provide a heuristic estimate of the number of distinct ecological groups needed to account for major patterns in functional potential across samples [51].

### Trait-based analysis of life-history strategies

To quantify life-history strategies using a top-down approach, we classified annotated functional genes into yield (Y), acquisition (A), and stress-tolerance (S) trait categories and calculated their relative abundances across successional time. Genomic traits representing Y, A, and S life-history strategy groups were defined from the literature (Supplementary Table S3), and the abundance of each group was calculated for every time point in each study. A and Y traits were defined using the gene lists reported in Wu *et al.* [52], and S traits were defined using stress-related KOs from the curated KO-to-trait mappings provided by microTrait [30]. KO abundances were normalized to the total number of quality-filtered reads, and summary values for each strategy group at each time point were generated by summing the normalized abundances. Finally, we assessed the Pearson correlation coefficient among the Y, A, and S abundances to evaluate potential trade-offs among life-history strategies.

## Results and discussion

The systematic review identified 71 studies fitting our criteria for functional primary succession (Supplementary Fig. S1). Of these, 54 examined the mammalian gut microbiome, leaving 17 studies from other systems, including soil (n = 8), biofilms (n = 5), phyllospheres (n = 1), insect gut (n = 1), animal nasopharynx (n = 1), and animal skin (n = 1). From these 17, eight studies met all methodological requirements for re-analysis (Table 1; see Materials and Methods for criteria), encompassing 507 samples. One of these studies [53] sampled three geographically distinct glacial forefields, which we treated as independent systems, resulting in a total of ten primary succession systems for comparative analysis. Here, “system” was defined as a unique successional sequence, including individual glacier forefields treated separately. There were five studies representing each of the autotrophic and heterotrophic exogenous succession types.

### Trends in functional diversity during primary succession differed by habitat

**Figure 1:**
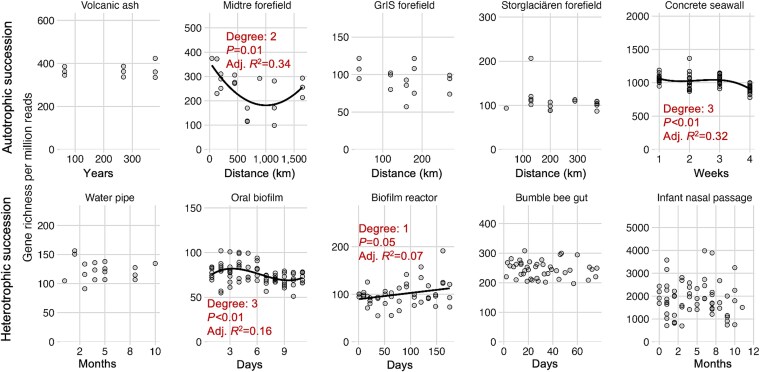
Functional gene richness during microbial primary succession. Each panel represents a distinct successional system. Autotrophic succession systems are shown in the top row, and heterotrophic systems are shown in the bottom row. Panels with regression lines indicate significant relationships between functional gene richness and successional time. GrIS: Greenland Ice Sheet. See Supplementary Table S4 for regression model results.

Significant relationships between functional gene richness and time were observed in four of ten systems (Fig. 1). Of the four systems, two were autotrophic and two were hetertrophic. In the Midtre Lovénbreen forefield, richness declined over time and subsequently increased 1500 km from the glacier front, as indicated by a quadratic fit. In contrast, both the oral biofilm and concrete seawall biofilm systems exhibited significant nonlinear declines in functional diversity over time. Only the biofilm reactor showed a linear increase in functional diversity.

These contrasting patterns in functional diversity may partly reflect differences in resource regimes and environmental constraints among habitats. In engineered biofilms, early colonizers can generate spatial heterogeneity and modify oxygen availability, shaping subsequent functional diversity [54, 55]. In both the oral biofilm and concrete seawall systems, oxygen concentrations decline as biofilms develop, potentially constraining aerobic metabolism and thereby limiting the range of viable metabolic strategies later in succession. Such patterns are consistent with theory predicting that resource gradients influence competitive dynamics and successional trajectories [56, 57]. As a sensitivity analysis, generalized additive mixed models with system as a random intercept indicated a weak, approximately linear effect of successional time on functional gene richness in autotrophic systems and no detectable nonlinear structure in heterotrophic systems (Supplementary Fig. S3), consistent with the limited and system-specific trends observed in the polynomial regressions. Together, these findings highlight how ecological context, sampling resolution, and the timing of observations influence our ability to detect and interpret functional succession across microbial systems.

### Community-average rRNA operon copy number and genome size differentiated primary succession in autotrophic systems

**Figure 2:**
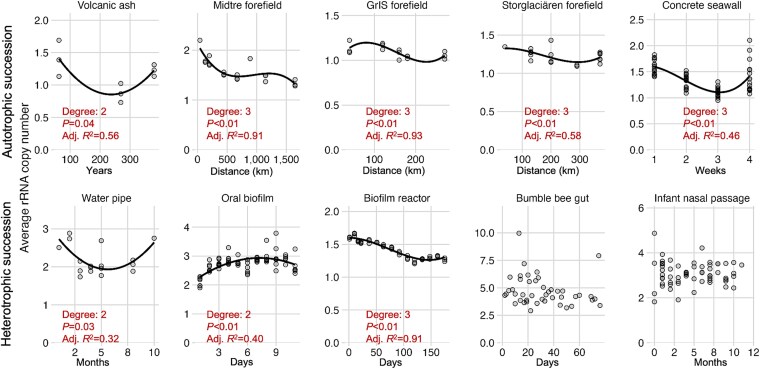
Average RRN during microbial primary succession. Each panel represents a distinct successional system. Autotrophic succession systems are shown in the top row and heterotrophic systems in the bottom row. Panels with regression lines indicate significant relationships between RRN and successional time. GrIS: Greenland Ice Sheet. See Supplementary Table S4 for regression model results.

RRN exhibited significant relationships with time in eight out of ten systems, including all five autotrophic and three heterotrophic systems (Fig. 2). All significant relationships, except for the oral biofilm microcosm, involved an initial, non-linear decline in RRN over time. No clear temporal trend in RRN was detected in the two host-associated heterotrophic systems (bumble bee gut and infant nasal passage; Fig. 2). GAMM results for RRN revealed modest nonlinear dynamics in autotrophic systems (EDF ≈ 3.1), consistent with initial declines followed by partial rebounds later in succession, while heterotrophic systems exhibited no consistent temporal signal (Supplementary Fig. S3).

The decrease in RRN aligns with previous studies of microbial primary succession, which have documented elevated RRN during early colonization phases [17, 25, 58]. Our findings corroborate this trend while avoiding a key limitation of previous approaches: earlier studies inferred genomic traits from 16S rRNA gene-derived taxonomic profiles, restricting analyses to taxa with available reference genomes—typically fast-growing, cosmopolitan organisms that are overrepresented in genome databases [59]. Here, our metagenomic approach estimates traits directly from functional gene abundances, avoiding the need for reference-based imputation. This allowed us to recover a more complete distribution of traits, including from slower-growing or less well-characterized taxa that may dominate in later succession. These results support a link between microbial succession and RRN and extend this pattern across a broader diversity of systems than previously described.

Nonlinear trajectories in RRN indicated by second- and third- order polynomial regression fits (Fig. 2) suggest that fast-growing taxa may not be restricted to early successional stages. As communities mature, increasing habitat heterogeneity and metabolic cross-feeding can generate microsites rich in diverse and labile organic carbon resources [60, 61], providing opportunities for taxa with high RRN to persist or re-emerge. Disturbance and turnover events, such as predation or rewetting, may further create transient resource surpluses that favor opportunists [62, 63]. In this context, coexistence mechanisms and niche diversification in late succession could support a mixture of low and high RRN taxa. Although such patterns may partly reflect the influence of dominant taxa or the limited temporal resolution of sampling (e.g. [64]), the ecological explanations point to dynamic resource environments that allow high-RRN taxa to remain important beyond initial colonization.

**Figure 3:**
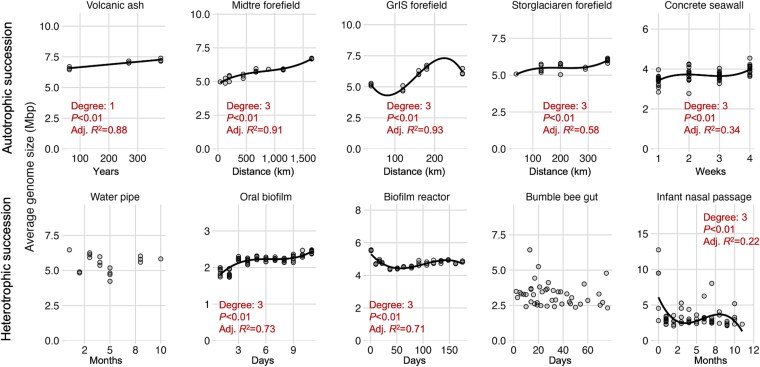
AGS during microbial primary succession. Each panel represents a distinct successional system. Autotrophic succession systems are shown in the top row and heterotrophic systems in the bottom row. Panels with regression lines indicate significant relationships between AGS and successional time. GrIS: Greenland Ice Sheet. See Supplementary Table S4 for regression model results.

AGS had a significant relationship with time in eight out of ten systems, including all five autotrophic and three heterotrophic systems (Fig. 3). Of these, AGS tended to increase over time in all five autotrophic systems and the oral biofilm, whereas it tended to decline over time in the biofilm reactor and infant nasal microbiome (Fig. 3). No significant trends detected in the water pipe or bumble bee gut. GAMMs supported a gradual, nonlinear increase in AGS over successional time in autotrophic systems (EDF ≈ 2.7), whereas heterotrophic systems showed no consistent temporal structure (EDF ≈ 1; Supplementary Fig. S3), aligning with the system-level patterns inferred from the polynomial regression.

Larger genomes are associated with greater regulatory and signaling capacity, and metabolic flexibility [22, 65], traits that may enhance ecological versatility under increasingly competitive or heterogenous conditions. In the context of succession, increasing AGS over time is consistent with a shift toward communities characterized by broader functional potential. This interpretation aligns with studies linking larger genomes to nutrient cycling capacity and resilience to environmental fluctuations [66], and with the idea that genome expansion carries metabolic costs [67] and is therefore more likely to be favored as resource availability increases. In three ecosystems (Midtre forefield, oral biofilm, and concrete seawall biofilm), increases in AGS coincided with declines in functional gene richness. Rather than indicating reduced functional capacity, this pattern may reflect increased functional redundancy, whereby larger genomes encode multiple genes providing capacity to perform similar or overlapping biochemical functions. Such redundancy can enhance ecological resilience and buffer communities against environmental fluctuations, even if the total number of unique functional categories declines [68]. This interpretation is consistent with the idea that later successional communities prioritize stability and competitive persistence over maximal functional breadth.

**Figure 4:**
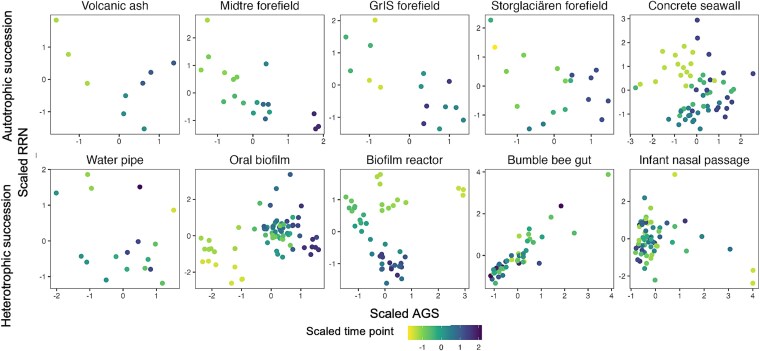
Covariation between average RRN and AGS across successional samples. Each panel represents a distinct successional system. Autotrophic succession systems are shown in the top row and heterotrophic systems in the bottom row. GrIS: Greenland Ice Sheet.

Systems hypothesized to undergo autotrophic succession, meaning they depend on in situ primary production for organic carbon, tended to exhibit a consistent trajectory from low AGS and high RRN in early succession toward higher AGS and lower RRN over time (Fig. 4, top row). Across autotrophic systems, RRN declined significantly with increasing AGS (linear mixed-effects model with system as a random intercept: β = −0.13 RRN per Mb, SE = 0.04, p < 0.01), and this association showed relatively limited between-system heterogeneity (system-level intercept SD = 0.20; residual SD = 0.21). This pattern indicates coordinated shifts in traits associated with growth rate and genomic versatility along successional gradients and likely reflects parallel responses of multiple traits to succession rather than a direct causal relationship between genome size and ribosomal copy number.

In contrast, heterotrophic systems exhibited more variable and system-specific trait trajectories (Fig. 4, bottom row). Across heterotrophic systems, RRN increased significantly with AGS (linear mixed-effects model with system as a random intercept: β = 0.17 RRN per Mb, SE = 0.05, *P* < .01), but this association was accompanied by substantial between-system heterogeneity (system-level intercept SD = 1.36; residual SD = 0.65). Individual systems illustrate this heterogeneity: the oral biofilm transitioned from low RRN and AGS to high values for both traits, whereas the water pipe system and the two host-associated microbiomes (bumble bee gut and infant nasal passage) showed little evidence of temporal trait trajectories. These systems are characterized by processes that can repeatedly restructure microbial communities, including disinfectant exposure in water pipe biofilms, host-mediated perturbations such as viral infections or antibiotic exposure in infant nasal microbiome, and continual reseeding of the bee gut microbiome from food sources. Such dynamics can promote trait stability rather than the directional trajectories observed in other systems.

In autotrophic systems, declining RRN and increasing AGS over time align closely with predictions of classical succession theory, in which early colonizers prioritize rapid growth and resource acquisition, whereas later communities favor efficiency and regulatory flexibility [28, 69]. This trajectory is consistent with theoretical expectations that as resources become depleted or more heterogeneous, competitive strategies increasingly replace opportunistic ones [56, 70], a pattern also observed in microbial biofilm succession where most early colonizers are replaced during community development [71]. By contrast, the greater variability observed in heterotrophic systems likely reflects differences in the composition, timing, or predictability of external organic carbon inputs. Because heterotrophic succession depends on detrital or allochthonous organic matter inputs, variation in substrate availability may favor divergent or overlapping strategies across successional stages. These context-dependent patterns emphasize how the type and dynamics of carbon inputs influence microbial life-history strategies and may explain why trait trajectories during succession are more consistent in autotrophic systems, where primary production imposes stronger constraints on community development, than in heterotrophic systems.

An exception to these cross-system patterns was observed in the biofilm reactor, where trait dynamics (declining AGS and RRN over time) contrasted with patterns in natural systems and likely reflected the influence of engineered, nitrogen-rich conditions. In this system, succession involved a transition from aerobic to anaerobic taxa as oxygen became depleted within the developing biofilm [54]. In nitrogen-replete environments, rapid biomass accumulation by early colonizers can promote the formation of anoxic microenvironments that enable the establishment of obligate anaerobes, illustrating environmental modification and facilitation during succession [56, 57, 70]. This example highlights how microbial succession can be strongly shaped by resource regimes and environmental modification within developing communities and underscores the need to expand succession frameworks to account for the diversity of substrate and energy flows that characterize microbial systems.

### Gene groups were functionally heterogeneous

**Figure 5:**
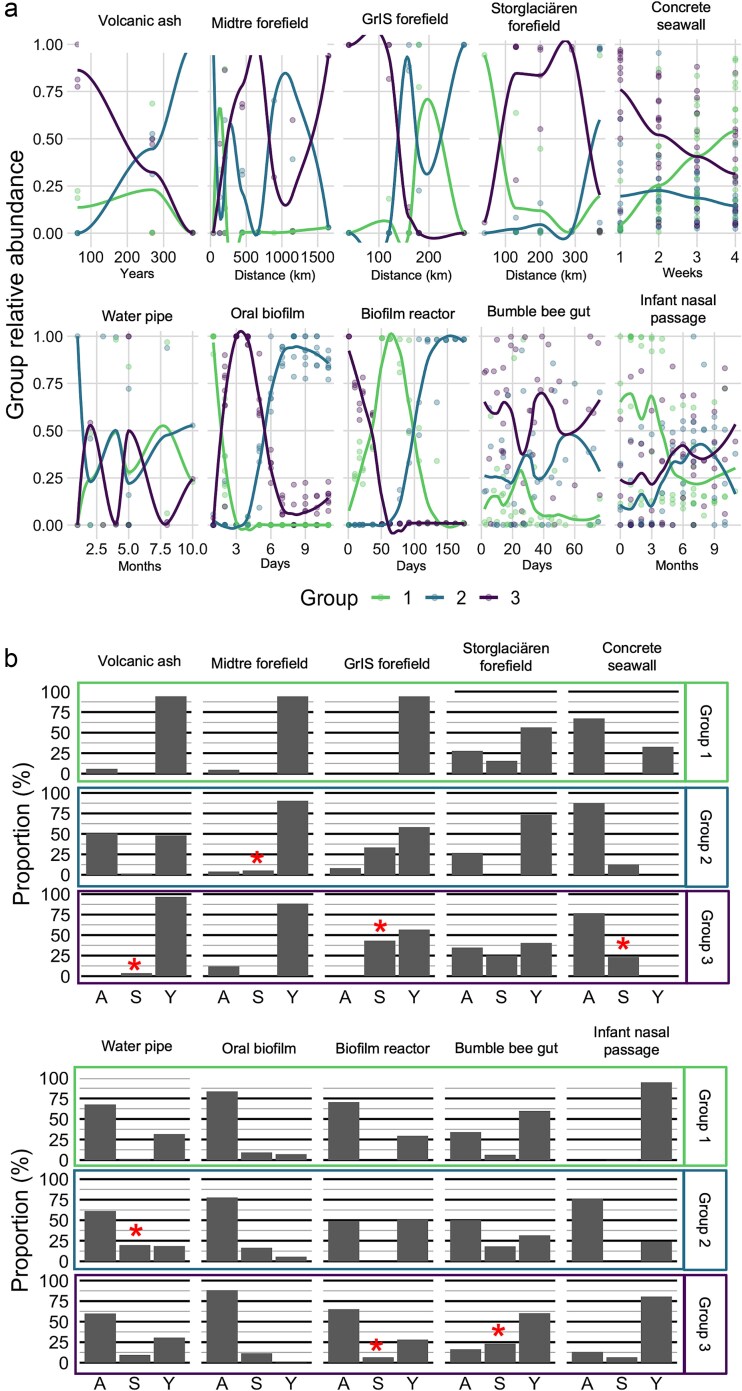
Temporal dynamics of functional gene grouping during microbial primary succession inferred using LDA. Each panel represents a distinct successional system. Autotrophic succession systems are shown in the top row and heterotrophic systems in the bottom row. For each study, LDA was applied to the sample × gene matrix and fit with three groups, such that each sample is represented by a mixture of up to three groups of genes. a) In each panel, points indicate the relative contribution (θ) of each group to individual samples across successional time. b) Columns summarize the life history strategy composition of the top 10 most strongly weighted genes in each group. Red stars indicate the group in which stress tolerance genes reached their greatest within-system proportion for each system. Life-history strategy assignments were based on KOs annotations using the published microTrait KO-to-trait mapping table [30], supplemented by manual classification where annotations were unavailable; individual gene classifications are provided in Supplementary Table S5. GrIS: Greenland Ice Sheet.

Topic modeling of life-history strategy genes revealed that several systems contained functional groups with distinct temporal dynamics (Fig. 5a; Supplementary Table S5). Across systems, the top 10 genes within each group accounted for a median of 97% (range 74%–100%) of total β weight, indicating that trait assignments represented the dominant functional signal within that functional gene group (topic). Overall, this method revealed no consistent trends in life history trait succession were observed across systems. However, in seven out of 10 systems, the group predominant in early succession (Fig. 5a) was the group in which genes associated with stress tolerance—including genes involved in cellular protection and environmental stress response such as chaperonins, compatible-solute transport systems, and multidrug resistance proteins—reached their greatest within-system proportion (Fig. 5b). This pattern aligns with the facilitation model of succession, in which early colonizers tolerate harsh or unstructured environments and modify them in ways that allow subsequent colonists to establish and later replace them [70]. These observations are also consistent with trait-based ecological theory predicting that stress-tolerance traits are favored under high abiotic constraint [72].

Three systems deviated from the pattern in which stress tolerance genes reached their greatest within-group proportion in early succession: Storglaciären forefield, the oral biofilm, and the infant nasal passage exhibited unique dynamics. In the oral biofilm, all three groups contained similar proportions of stress tolerance genes (Fig. 5b); however, the types of stress tolerance genes differed. Early succession stress tolerance genes were related to antibiotic production (Group 1; Supplementary Table S5), whereas later-stage stress tolerance genes were associated with extracellular polysaccharide production and biofilm formation (Group 2; Supplementary Table S5). Given that biofilm formation is intrinsic to community assembly in this environment [73], it may not represent stress adaptation per se. When interpreted in this light, the oral biofilm may still follow the broader trend of early-stage stress adaptation. In contrast, the infant nasal microbiome showed an increase in stress tolerance traits over time (Fig. 5). One possible explanation is that early colonization took place in a relatively hospitable environment with abundant carbon substrates, low microbial density, underdeveloped immune defenses, and limited competition, whereas later stages were shaped by increasing host immune activity, environmental exposure, and microbial interactions that selected for stress-related functions [74–76]. In Storglaciären forefield, the group with the greatest abundance in early succession contained a greater proportion of resource use genes, and the group with more stress tolerance genes became more abundant at the next time point (Fig. 5).

Patterns of functional group stability varied markedly across systems. Of all systems, the bumble bee gut exhibited the greatest degree of stability in LDA gene group composition throughout succession (Fig. 5a), consistent with the original study’s observation of a conserved, socially transmitted core host microbiota [77]. In contrast, the glacier forefield systems and water pipe biofilm lacked similar stability and did not exhibit clear directional change (Fig. 5a). In the bumble bee gut, strong host filtering and colony-level transmission may contribute to functional stability over time [78]. By comparison, the glacier forefields and water pipe system likely experienced greater environmental heterogeneity across sampling gradients, potentially obscuring directional trends in trait composition. For glacier forefields, this heterogeneity could reflect variation in microtopography, hydrology, or soil development stage [79]. In the water pipe system, the original study reported strong seasonal variation in biofilm composition [80], suggesting that temporal fluctuations in environmental conditions may have masked successional trends.

**Figure 6:**
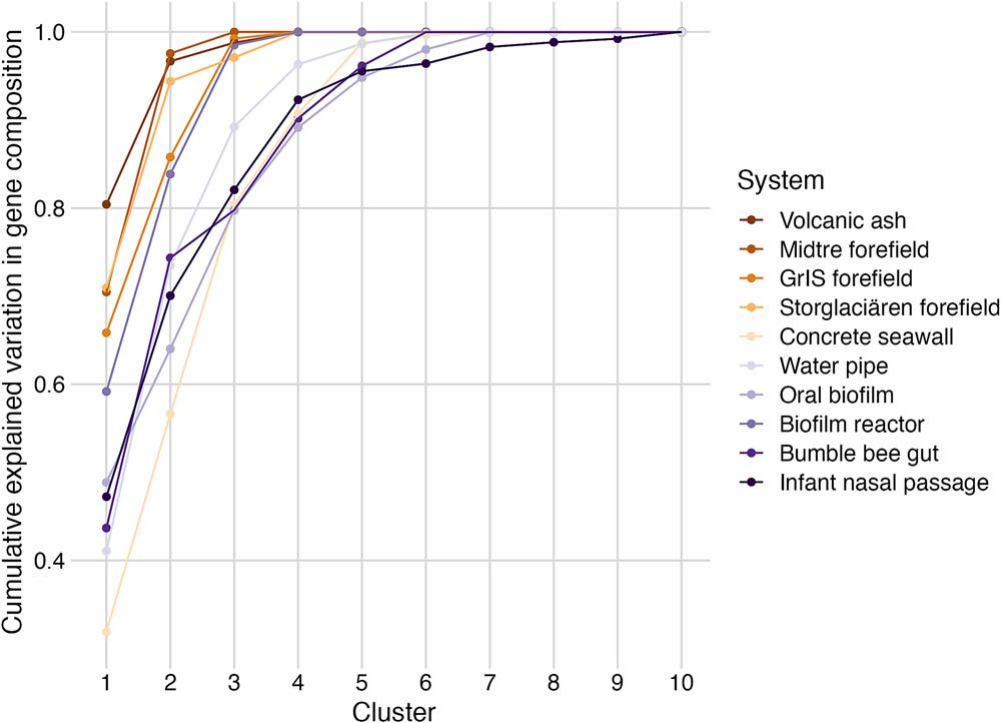
Cumulative contribution of latent ecological groups to variation in gene abundances across samples. Each curve point shows the cumulative proportion of total gene abundance variation explained as additional LDA-inferred groups are included, based on average group proportions (θ) inferred from a LDA model. This group number indicates how many distinct underlying patterns are needed to capture the major trends in gene composition across the dataset. Autotrophic succession systems are shown in warm colors heterotrophic systems are in cool colors. GrIS: Greenland Ice Sheet.

Across studies, the three LDA-identified functional gene groups explained 80–100% of the variation in gene composition, while two groups explained 70–99%, with higher variation described in autotrophic than heterotrophic systems (Fig. 6). This suggests that microbial functional succession within individual systems can often be captured by a small number of co-occurring gene groups, as has been noted elsewhere [81–83]. The fact that a small number of groups accounted for most of the functional variation supports the utility of topic modeling for identifying dominant ecological strategies in microorganisms without requiring strict one-to-one mapping between genes and traits, extending the application of LDA to biodiversity data beyond species assemblages [84]. The LDA groups tended to be functionally heterogeneous, reinforcing the idea that microbial succession may involve flexible combinations of traits rather than discrete, single-strategy groups (i.e. Y–A–S). This also aligns with the idea that microbial life-history strategies emerge from coordinated trade-offs across multiple ecological functions, which may vary in their relative importance across diverse environments with differing resource constraints [85, 86]. Furthermore, when the number of groups was reduced, autotrophic systems tended to retain more explained variation than heterotrophic systems (Fig. 6), consistent with the hypothesis that higher chemical diversity associated with complex organic matter inputs can drive more varied functional outcomes during microbial succession, as has been observed in soil, aquatic, and engineered systems [87–91].

### Yield- and stress-tolerance genes tracked total gene counts

We next explored whether microbial strategies during succession could be characterized by trade-offs among yield, resource acquisition, and stress tolerance, as proposed in the Y–A–S life-history framework [29]. Across systems, we found that the relative abundance of yield- and stress-related genes were highly positively correlated (Pearson’s *r* ranged from 0.84–0.99), as well as positively correlated with total functional gene counts (Pearson’s *r* ranged from 0.95–0.99 for yield-related genes), suggesting that microbial life-history strategies during succession reflect coupled adaptations to stress and growth. This relationship was not explained by AGS or sequencing depth, indicating a biologically meaningful pattern rather than a technical artifact. One possible interpretation is that in resource-limited or environmentally stressful conditions, traits that promote efficient biomass production (Y) and stress resilience (S) are co-selected, enabling persistence under challenging conditions such as during early succession. Consistent with this pattern, the functional groups identified through topic modeling did not correspond to discrete Y, A, or S strategies, but instead contained mixtures of genes associated with multiple life-history traits. This pattern suggests that yield and stress tolerance strategies may often operate together during microbial succession, rather than as separate strategies. While recent trait-based ordination studies have supported a three-axis model across broad environmental gradients in soils [33, 34], our findings highlight that the importance of each trait axis may vary by context during succession—consistent with earlier calls to tailor trait-based models to specific ecological settings [32, 92].

Although our trait-based approach identified consistent patterns across systems, several limitations warrant consideration. First, many studies included in the analysis began sampling only after initial colonization, potentially missing rapid or transient dynamics that define earliest succession. These early phases may be marked by sharp shifts in functional diversity or trait dominance that attenuate over time—a pattern observed in both macro- and microbial systems [9, 25, 93]. Second, the framework relies on a limited set of broad traits that enable cross-system comparison but may overlook finer-scale or system-specific functions—such as host-mediated selection in host microbiomes or spatial structuring in biofilms. For instance, in the bumble bee gut system, a species turnover from *Gilliamella* to *Schmidhempelia* may reflect a trait shift relevant to host immunity or metabolism [77] that was not captured in our selected trait set. These findings underscore the need to balance generalizable trait frameworks with ecological specificity, and to prioritize study designs that capture the earliest stages of microbial community assembly, even though low biomass may pose a technical challenge. Finally, consideration of microbial Eukaryotes, which may influence microbial community assembly through provision of carbon and nutrients, structure, or through competition and predation, could provide a valuable perspective [63, 73, 100, 101]. Although we removed genes exclusive to Eukaryotes, this would not account for genes common to both Prokaryotes and Eukaryotes and could therefore introduce minor ambiguity; however, the vast majority of genes captured in our analyses are well established as prokaryotic in origin, supporting our use of the term “microbes” to refer specifically to bacterial and Archaeal communities in this study.

## Conclusions

The clearest signal of microbial primary succession was a consistent decline in RRN accompanied by an increase in AGS in autotrophic systems, whereas heterotrophic systems exhibited more variable and context-dependent trajectories. Interpreted through an r/*K* strategy lens, these results indicate a shift from growth-rate–associated strategies toward greater resource-use efficiency and genomic versatility as autotrophic communities mature. Our findings extend previous work showing that rRNA operon copy number tracks microbial life-history strategies across successional gradients [25, 58], demonstrating that RRN consistently declines during primary succession in autotrophic systems. In parallel, increasing AGS supports the prediction that later successional communities invest in expanded regulatory and metabolic versatility [22, 65, 66].

By contrast, patterns derived from the Y–A–S framework were less uniform across systems. Although LDA identified functional groupings associated with early, middle, and late successional stages, no single Y–A–S axis exhibited a universal directional trajectory over successional time. Instead, yield- and stress-related genes were strongly correlated with one another and with overall functional gene counts. These patterns suggest that microbial life-history trade-offs during succession may not resolve cleanly into discrete Y, A, and S strategies, but rather reflect coupled and context-dependent trait syndromes.

Together, these findings show that r/*K*-associated traits (RRN and AGS) provide a robust and comparable signal of successional direction, particularly in autotrophic systems. These axes of microbial strategy provide a tractable foundation for cross-system comparisons and suggest that universal trait dimensions capture predictable successional trajectories in autotrophic systems, whereas heterotrophic succession requires closer attention to resource dynamics and ecological context.

## Supplementary Material

Supplementary_material_for_Cagle_et_al_Revised_wrag134

Supplementary_table_S3_YAS_traits_merged_wrag134

Supplementary_table_5_LDA_genes_results_wrag134

## Data Availability

The datasets analyzed during the current study are publicly available from the SRA (https://www.ncbi.nlm.nih.gov/sra) and ENA (https://www.ebi.ac.uk/ena) databases under the data identification codes listed in Table 1.
