# Supplementary material for: Carbon regime structures functional trait trajectories during primary succession in microorganisms
Source: ISME J. 2026 May 23;20(1):wrag134. doi: 10.1093/ismejo/wrag134 (PMC13374867; doi:10.1093/ismejo/wrag134)
Supplement: Supplementary_material_for_Cagle_et_al_Revised_wrag134 [file supplementary_material_for_cagle_et_al_revised_wrag134.pdf]

Supplementary Data and Methods for: Carbon regime structures functional trait trajectories during microbial primary succession

Grace A. Cagle[1], Benjamin Baiser[2], Jessica R. Bernardin[3], Leonora S. Bittleston[3], Erica B. Young[4], Sarah M. Gray[5], Zachary B. Freedman*[1]

[1]Department of Soil and Environmental Science, University of Wisconsin-Madison, Madison, Wisconsin, USA; [2]Department of Wildlife Ecology and Conservation, University of Florida, Gainesville, Florida, USA; [3]Department of Biological Sciences, Boise State University, Boise, ID USA; [4]Department of Biological Sciences and School of Freshwater Sciences, University of Wisconsin-Milwaukee, Milwaukee, Wisconsin, USA; [5]Department of Biology-Ecology and Evolution, University of Fribourg, Fribourg, Switzerland

*__Correspondence__: Zachary B. Freedman—zfreedman@wisc.edu; 1525 Observatory Drive, Madison, WI, 53705

Detailed methods for systematic literature review. The search string was developed by building out three key components required for the study, which were 1) bacteria, 2) primary succession, and 3) metagenomic sequencing (Supplementary table 2). The search terms were developed collaboratively by all co-authors and with consultation from a subject-matter expert librarian at University of Wisconsin–Madison. A single author (GC) screened each record independently. Automation tools within the Rayyan software were used to identify duplicates and identify keywords (Ouzzani et al., 2016) and ChatGPT was used to summarize abstracts using a python script.

Detailed methods for the selection of studies for re-analysis. Of the 17 non-mammal gut studies that were candidates for reanalysis, 12 met our methodological requirement for reanalysis of being registered through the SRA or ENA databases. In the case that the data were publicly available, but sample providence could not be determined, the corresponding author of the study was asked via email to provide within three weeks the information necessary to identify the samples before the study was excluded. We were unable to obtain data from four of the 12 candidate studies for the following reasons: the data were missing or failed to upload correctly (Li et al., 2022; Feng et al., 2023; Bech et al., 2024), or the data lacked sample providence information (Chaudhary et al., 2023). At least one attempt to contact the corresponding author was made in all cases for which the data could not be obtained.

Detailed methods for bioinformatics. Metagenomic sequencing reads were downloaded using SRA-tools (https://github.com/ncbi/sra-tools) and the Anvio code base (Eren et al., 2020). Reads were subsequently quality filtered using BBTools (Bushnell et al., 2017), including adapter trimming, the removal of contaminating human sequences, and, where relevant, the removal of host genome sequences. Following quality control, reads were deduplicated with BBTools, and their original copy numbers were retained to enable accurate quantification of gene abundance. Coding regions were identified by predicting open-reading frames using FragGeneScanRs (Van der Jeugt et al., 2022). To ensure accurate functional assignment and quantification, the annotation with the highest HMMER score was selected for each coding region, and gene counts were calculated by multiplying each gene's abundance by its retained copy number. Singleton genes were discarded. Relative abundance for genes in each sample was calculated as the gene's abundance divided by the total number of reads in the sample. To obtain an integer value required for LDA, relative abundance values were multiplied by a large constant (e.g., 1×10^6).

Detailed methods for sample filtering. Several samples were excluded from downstream analysis to ensure data quality and sufficient replication. For the infant nasal microbiome dataset (Accorsi et al., 2020), two samples exhibiting gene counts approximately 100-fold greater or markedly lower than other samples were removed (SRR11262358, SRR11262573). In the bumble bee gut microbiome dataset (Hammer et al., 2023), time points beyond 90 days were omitted due to low replication (SRR19756540, SRR19756542). Similarly, control samples from the concrete seawall biofilm study (Summers et al., 2022) were excluded (SRR15682911, SRR15682875, SRR15682900, SRR15682874, SRR15682933, SRR15682889, SRR15682922, SRR15682878, SRR15682945, SRR15682944, SRR15682943, SRR15682942). Lastly, in the glacier forefield datasets from Nash et al. (2018), two sequencing runs with low read counts (ERR4837119, ERR4837121) and five Greenland Ice Sheet (GrIS) forefield samples located more than 3,000 km from the glacier forefront were removed due to insufficient replication (ERR4837100, ERR4837101, ERR4837102, ERR4837103, ERR4837104).

References

Accorsi, E.K., Franzosa, E.A., Hsu, T., Joice Cordy, R., Maayan-Metzger, A., Jaber, H., Reiss-Mandel, A., Kline, M., DuLong, C., Lipsitch, M., Regev-Yochay, G., Huttenhower, C., 2020. Determinants of Staphylococcus aureus carriage in the developing infant nasal microbiome. Genome Biology 21, 301. https://doi.org/10.1186/s13059-020-02209-7

Bech, P.K., Jarmusch, S.A., Rasmussen, J.A., Limborg, M.T., Gram, L., Henriksen, N.N.S.E., 2024. Succession of microbial community composition and secondary metabolism during marine biofilm development. ISME Communications 4, ycae006. https://doi.org/10.1093/ismeco/ycae006

Bushnell, B., Rood, J., Singer, E., 2017. BBMerge – Accurate paired shotgun read merging via overlap. PLOS ONE 12, e0185056. https://doi.org/10.1371/journal.pone.0185056

Chaudhary, P.P., Myles, I.A., Zeldin, J., Dabdoub, S., Deopujari, V., Baveja, R., Baker, R., Bengtson, S., Sutton, A., Levy, S., Hourigan, S.K., 2023. Shotgun metagenomic sequencing on skin microbiome indicates dysbiosis exists prior to the onset of atopic dermatitis. Allergy 78, 2724–2731. https://doi.org/10.1111/all.15806

Eren, A.M., Kiefl, E., Shaiber, A., Veseli, I., Miller, S.E., Schechter, M.S., Fink, I., Pan, J.N., Yousef, M., Fogarty, E.C., Trigodet, F., Watson, A.R., Esen, Ö.C., Moore, R.M., Clayssen, Q., Lee, M.D., Kivenson, V., Graham, E.D., Merrill, B.D., Karkman, A., Blankenberg, D., Eppley, J.M., Sjödin, A., Scott, J.J., Vázquez-Campos, X., McKay, L.J., McDaniel, E.A., Stevens, S.L.R., Anderson, R.E., Fuessel, J., Fernandez-Guerra, A., Maignien, L., Delmont, T.O., Willis, A.D., 2021. Community-led, integrated, reproducible multi-omics with anvi'o. Nat Microbiol 6, 3–6. https://doi.org/10.1038/s41564-020-00834-3

Feng, M., Varliero, G., Qi, W., Stierli, B., Edwards, A., Robinson, S., van der Heijden, M.G.A., Frey, B., 2023. Microbial dynamics in soils of the Damma glacier forefield show succession in the functional genetic potential. Environmental Microbiology 25, 3116–3138. https://doi.org/10.1111/1462-2920.16497

Hammer, T.J., Easton-Calabria, A., Moran, N.A., 2023. Microbiome assembly and maintenance across the lifespan of bumble bee workers. Molecular Ecology 32, 724–740. https://doi.org/10.1111/mec.16769

Li, Y., Gao, P., Sun, X., Li, B., Guo, L., Yang, R., Su, X., Gao, W., Xu, Z., Yan, G., Wang, Q., Sun, W., 2022. Primary Succession Changes the Composition and Functioning of the Protist Community on Mine Tailings, Especially Phototrophic Protists. ACS Environ. Au 2, 396–408. https://doi.org/10.1021/acsenvironau.1c00066

Nash, M.V., Anesio, A.M., Barker, G., Tranter, M., Varliero, G., Eloe-Fadrosh, E.A., Nielsen, T., Turpin-Jelfs, T., Benning, L.G., Sánchez-Baracaldo, P., 2018. Metagenomic insights into diazotrophic communities across Arctic glacier forefields. FEMS Microbiology Ecology 94. https://doi.org/10.1093/femsec/fiy114

Ouzzani, M., Hammady, H., Fedorowicz, Z., Elmagarmid, A., 2016. Rayyan—a web and mobile app for systematic reviews. Systematic Reviews 5, 210. https://doi.org/10.1186/s13643-016-0384-4

Summers, S., Pek, Y.S., Vinod, D.P., McDougald, D., Todd, P.A., Birch, W.R., Rice, S.A., 2022. Bacterial biofilm colonization and succession in tropical marine waters are similar across different types of stone materials used in seawall construction. Frontiers in Microbiology 13, 1–13. https://doi.org/10.3389/fmicb.2022.928877

Van der Jeugt, F., Dawyndt, P., Mesuere, B., 2022. FragGeneScanRs: faster gene prediction for short reads. BMC Bioinformatics 23, 198. https://doi.org/10.1186/s12859-022-04736-5

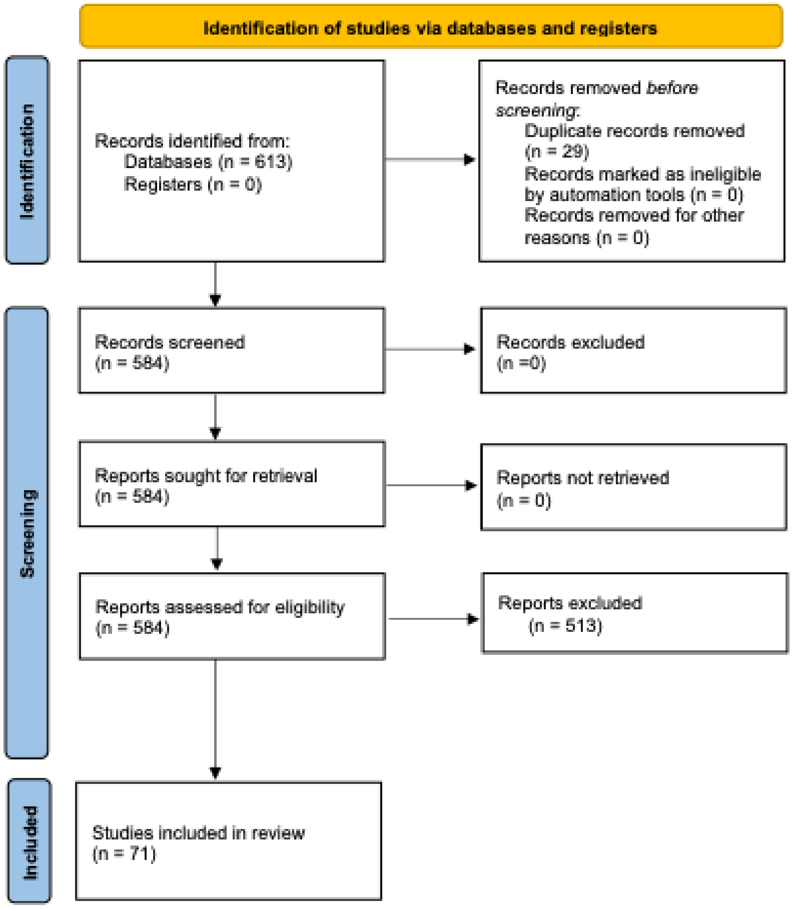

Supplementary Figure S1. PRISMA flow diagram showing the identification, screening, and inclusion of studies in the systematic literature review.

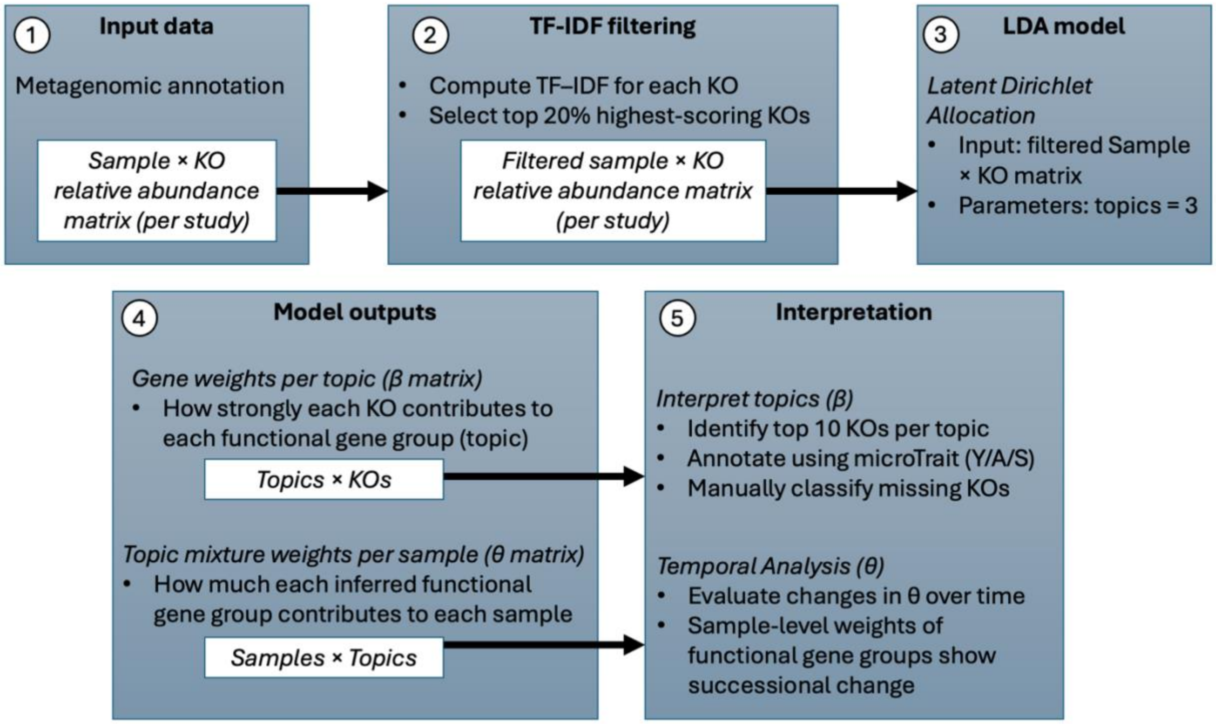

Supplementary Figure S2. Workflow for LDA-based identification of functional gene groups during microbial succession. Schematic overview of the topic-modeling analysis. (1) Within each study, metagenomic reads were annotated to KEGG orthologs (KOs) and converted to sample × gene relative abundance matrices. (2) To reduce noise and emphasize informative variation, term frequency–inverse document frequency (TF–IDF) scores were calculated and the top 20% of KOs were retained. (3) Latent Dirichlet Allocation (LDA), an unsupervised topic-modeling approach, was applied with the number of topics fixed at three to represent hypothesized life-history strategy trade-offs (growth yield [Y], resource acquisition [A], and stress tolerance [S]). (4) Model outputs included θ (topic mixture weights per sample), describing the relative contribution of each inferred functional gene group to each sample, and β (gene weights per topic), describing the contribution of each KO to its corresponding gene group. (5) Temporal trends in θ were used to evaluate how functional gene group weights shifted during succession, and the top 10 KOs in each β-distribution were microTrait life-history strategy assignments, supplemented by manual classification for KOs lacking annotations.

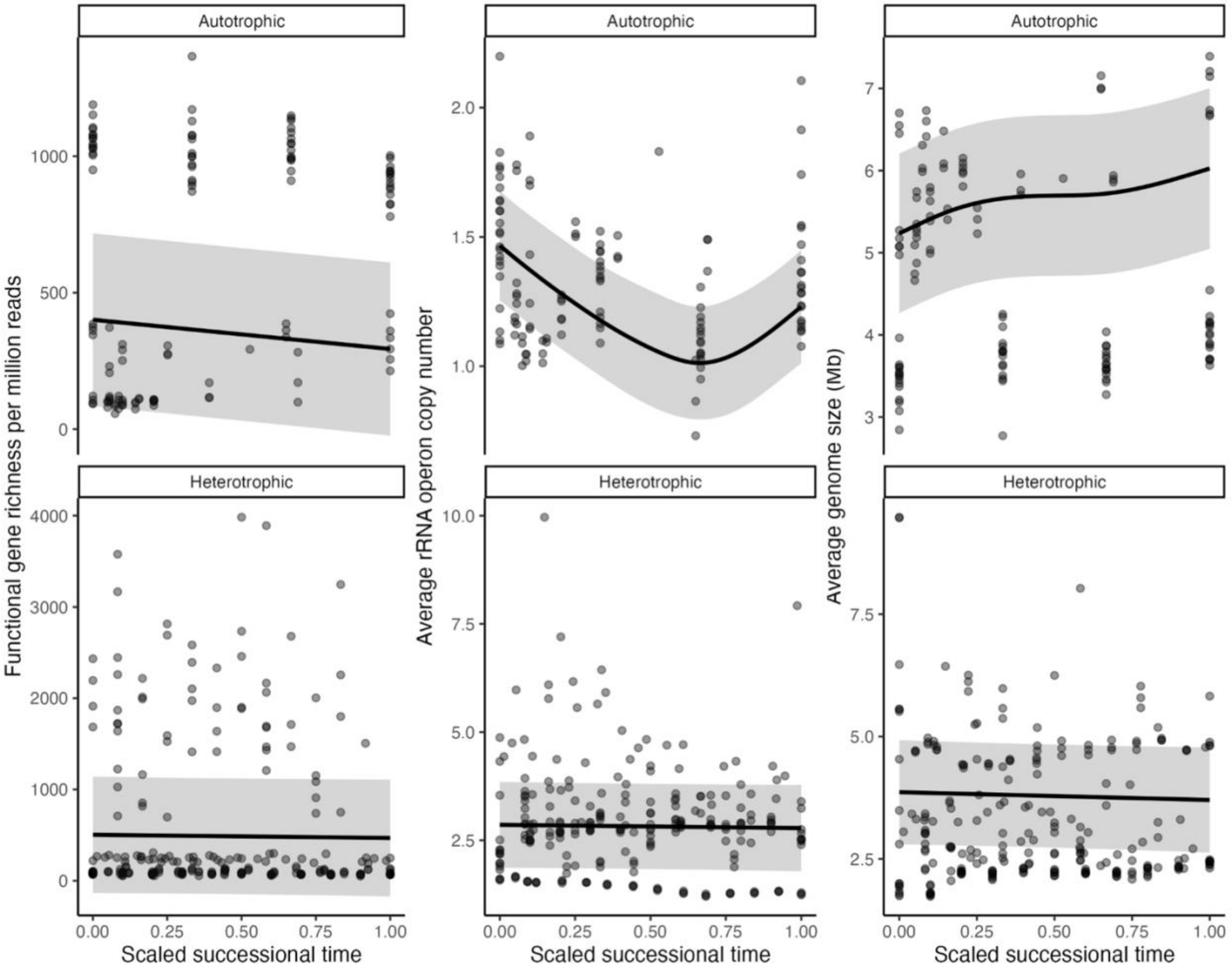

Supplementary Figure S3. Generalized additive mixed models (GAMMs) describing temporal trends across autotrophic (top) and heterotrophic (bottom) successional systems. GAMMs were fit for A) Functional gene richness, B) Average genome size (AGS), and C) Average rRNA operon copy number (RRN) during primary succession. Separate models were fit for autotrophic and heterotrophic systems, with system included as a random intercept. Points represent observed metagenomic samples. Solid lines indicate the fitted smooth term for time (scaled within each system), and shaded ribbons represent ±95% confidence intervals around the smooth. These models were conducted as a sensitivity analysis to evaluate whether nonlinear temporal patterns were detectable across systems without imposing system-specific polynomial forms.

Supplementary Table S1. PRISMA checklist documenting reporting standards followed for the systematic literature review.

Section and Topic	Item #	Checklist item	Location where item is reported
**TITLE**			
Title	1	Identify the report as a systematic review.	Title
**ABSTRACT**			
Abstract	2	See the PRISMA 2020 for Abstracts checklist.	Abstract
**INTRODUCTION**			
Rationale	3	Describe the rationale for the review in the context of existing knowledge.	Introduction
Objectives	4	Provide an explicit statement of the objective(s) or question(s) the review addresses.	Introduction
**METHODS**			
Eligibility criteria	5	Specify the inclusion and exclusion criteria for the review and how studies were grouped for the syntheses.	Methods
Information sources	6	Specify all databases, registers, websites, organisations, reference lists and other sources searched or consulted to identify studies. Specify the date when each source was last searched or consulted.	Supplementary table 2
Search strategy	7	Present the full search strategies for all databases, registers and websites, including any filters and limits used.	Supplementary table 2
Selection process	8	Specify the methods used to decide whether a study met the inclusion criteria of the review, including how many reviewers screened each record and each report retrieved, whether they worked independently, and if applicable, details of automation tools used in the process.	Methods and Supplementary material
Data collection process	9	Specify the methods used to collect data from reports, including how many reviewers collected data from each report, whether they worked independently, any processes for obtaining or confirming data from study investigators, and if applicable, details of automation tools used in the process.	Supplementary material
Data items	10a	List and define all outcomes for which data were sought. Specify whether all results that were compatible with each outcome domain in each study were sought (e.g. for all measures, time points, analyses), and if not, the methods used to decide which results to collect.	Methods
	10b	List and define all other variables for which data were sought (e.g. participant and intervention characteristics, funding sources). Describe any assumptions made about any missing or unclear information.	NA
Study risk of bias	11	Specify the methods used to assess risk of bias in the included studies, including details of the tool(s)	NA

Section and Topic	Item #	Checklist item	Location where item is reported
assessment		used, how many reviewers assessed each study and whether they worked independently, and if applicable, details of automation tools used in the process.	
Effect measures	12	Specify for each outcome the effect measure(s) (e.g. risk ratio, mean difference) used in the synthesis or presentation of results.	Methods
Synthesis methods	13a	Describe the processes used to decide which studies were eligible for each synthesis (e.g. tabulating the study intervention characteristics and comparing against the planned groups for each synthesis (item #5)).	Methods
	13b	Describe any methods required to prepare the data for presentation or synthesis, such as handling of missing summary statistics, or data conversions.	Methods and supplementary material
	13c	Describe any methods used to tabulate or visually display results of individual studies and syntheses.	Methods
	13d	Describe any methods used to synthesize results and provide a rationale for the choice(s). If meta-analysis was performed, describe the model(s), method(s) to identify the presence and extent of statistical heterogeneity, and software package(s) used.	Methods
	13e	Describe any methods used to explore possible causes of heterogeneity among study results (e.g. subgroup analysis, meta-regression).	NA
	13f	Describe any sensitivity analyses conducted to assess robustness of the synthesized results.	NA
Reporting bias assessment	14	Describe any methods used to assess risk of bias due to missing results in a synthesis (arising from reporting biases).	NA
Certainty assessment	15	Describe any methods used to assess certainty (or confidence) in the body of evidence for an outcome.	NA
**RESULTS**			
Study selection	16a	Describe the results of the search and selection process, from the number of records identified in the search to the number of studies included in the review, ideally using a flow diagram.	Supplementary figure 1
	16b	Cite studies that might appear to meet the inclusion criteria, but which were excluded, and explain why they were excluded.	Supplementary material
Study characteristics	17	Cite each included study and present its characteristics.	Results; Table 1
Risk of bias in studies	18	Present assessments of risk of bias for each included study.	NA
Results of individual	19	For all outcomes, present, for each study: (a) summary statistics for each group (where appropriate) and (b) an effect estimate and its precision (e.g. confidence/credible interval), ideally using structured	Results; Table 1

Section and Topic	Item #	Checklist item	Location where item is reported
studies		tables or plots.	
Results of syntheses	20a	For each synthesis, briefly summarise the characteristics and risk of bias among contributing studies.	NA
	20b	Present results of all statistical syntheses conducted. If meta-analysis was done, present for each the summary estimate and its precision (e.g. confidence/credible interval) and measures of statistical heterogeneity. If comparing groups, describe the direction of the effect.	Results
	20c	Present results of all investigations of possible causes of heterogeneity among study results.	Results
	20d	Present results of all sensitivity analyses conducted to assess the robustness of the synthesized results.	NA
Reporting biases	21	Present assessments of risk of bias due to missing results (arising from reporting biases) for each synthesis assessed.	NA
Certainty of evidence	22	Present assessments of certainty (or confidence) in the body of evidence for each outcome assessed.	NA
**DISCUSSION**			
Discussion	23a	Provide a general interpretation of the results in the context of other evidence.	Discussion
	23b	Discuss any limitations of the evidence included in the review.	Discussion
	23c	Discuss any limitations of the review processes used.	
	23d	Discuss implications of the results for practice, policy, and future research.	Discussion
**OTHER INFORMATION**			
Registration and protocol	24a	Provide registration information for the review, including register name and registration number, or state that the review was not registered.	Not registered
	24b	Indicate where the review protocol can be accessed, or state that a protocol was not prepared.	NA
	24c	Describe and explain any amendments to information provided at registration or in the protocol.	NA
Support	25	Describe sources of financial or non-financial support for the review, and the role of the funders or sponsors in the review.	Funding statement
Competing interests	26	Declare any competing interests of review authors.	Conflict of Interest Statement
Availability of data, code and other materials	27	Report which of the following are publicly available and where they can be found: template data collection forms; data extracted from included studies; data used for all analyses; analytic code; any other materials used in the review.	Data availability statement

Supplementary Table S2. Details of the literature search strategy used in the systematic review, reported in accordance with PRISMA guidelines. Searches were conducted on 04-04-2024

String	Target	Hits
(((ALL=(metagenom* or "shotgun sequencing")) AND TS=(bacteria or microb*)) AND TS=("primary succession" or "pioneer communit*" or "temporal colonization" or "priority effect*") AND TS=("time-series" or day* or week* or month* or year* or longitudinal or succession or seasonal*)) NOT DT=(Review or Book Chapter)	Primary succession	17
(((ALL=(metagenom*)) AND TS=(bacteria or microb*)) AND TS=(proglacial OR glacier* AND (forefield* or reced* or retreat*)) AND TS=("time-series" or day* or week* or month* or year* or longitudinal or succession or seasonal*)) NOT DT=(Review or Book Chapter)	Glacier retreat	12
(((ALL=(metagenom*)) AND TS=(bacteria or microb*)) AND TS=("volcanic ash" or "lava" or "volcanic eruption" or "volcanic deposit*") AND TS=("time-series" or day* or week* or month* or year* or longitudinal or succession or seasonal*)) NOT DT=(Review or Book)	Volcanic eruption	3
(((ALL=(metagenom*)) AND TS=(bacteria or microb*)) AND TS=(phyllosphere or "tissue age" or "leaf age") AND TS=("time-series" or day* or week* or month* or year* or longitudinal or succession or seasonal*)) NOT DT=(Review or Book or Proceeding Paper)	Leaf emergence	33
(((ALL=(metagenom* or "shotgun sequencing")) AND TS=(bacteria or microb*)) AND TS=(newborn or hatch* or neonat* or infant*) AND TS=("time-series" or day* or week* or month* or year* or longitudinal or succession or seasonal*)) NOT DT=(Review or Book Chapter)	Newborns	409
(((ALL=(metagenom* or "shotgun sequencing")) AND TS=(bacteria or microb*)) AND TS=("germ-free" or "gnotobiotic") AND TS=("time-series" or day* or week* or month* or year* or longitudinal or succession or seasonal*)) NOT DT=(Review)	Germ-free life	57
(((ALL=(metagenom*)) AND TS=(bacteria or microb*)) AND ALL=("pitcher plant" or "Sarracenia*") AND TS=("time-series" or day* or week* or month* or year* or longitudinal or succession or seasonal*)) NOT DT=(Review)	Pitcher plant	1
(((ALL=(metagenom*)) AND TS=(bacteria or microb*)) AND TS=("biofouling") AND TS=("time-series" or day* or week* or month* or year* or longitudinal or succession or seasonal*)) NOT DT=(Review)	Pipe and tube biofouling	10
(((ALL=(metagenom*)) AND TS=(bacteria or microb*)) AND TS=("biofilm formation" or "biofilm development") AND TS=("time-series" or day* or week* or month* or year* or longitudinal or succession or seasonal*)) NOT DT=(Review)	Biofilms	67
(((ALL=(metagenom*)) AND TS=(bacteria or microb*)) AND TS=("clean room" or "controlled environment") AND TS=("time-series" or day* or week* or month* or year* or longitudinal or succession or seasonal*)) NOT DT=(Review)	Controlled environment	4

Supplementary Table S3. Genes identified from the literature as representative of yield, resource acquisition, and stress-tolerance strategies. A and Y traits were defined using the gene lists reported in Wu et al. (2025), and S traits were defined using stress-related KOs from the curated KO-to-trait mappings provided by microTrait (Karaoz and Brodie, 2022).

(Separate Excel file)

Supplementary Table S4. The results of fitting polynomial regression models for functional gene richness, rRNA operon copy number and genome size.

**Functional gene richness**					
	model	*P*-value	Adjusted R^2	BIC	Between-model Chi-square test significance
Volcanic ash	y ~ x	p<0.01	0.88	37.77	ns
	y ~ x + x2	p<0.01	0.87	37.33	
	y ~ x + x2 + x3	* insufficient data points to fit model			
Glacier forefield	y ~ x	p<0.01	0.23	736.32	ns
	y ~ x + x2	p<0.01	0.22	740.15	
	y ~ x + x2 + x3	p<0.01	0.21	743.63	
Concrete seawall	y ~ x	p<0.01	0.17	510.18	
	y ~ x + x2	p<0.01	0.20	510.72	.
	y ~ x + x2 + x3	p<0.01	0.26	509.03	*
Water pipe	y ~ x	p=0.90	<0.01	212.62	
	y ~ x + x2	p=0.01	0.38	205.18	**
	y ~ x + x2 + x3	p=0.04	0.33	207.90	
Bumble bee gut	y ~ x	p=0.22	0.01	461.57	
	y ~ x + x2	p=0.43	<0.01	465.12	
	y ~ x + x2 + x3	p=0.56	<0.01	468.51	
Teeth	y ~ x	p=0.04	0.04	1004.38	
	y ~ x + x2	p=0.01	0.09	1003.96	*
	y ~ x + x2 + x3	p<0.01	0.15	1001.96	*
Biofilm reactor	y ~ x	p=0.08	0.05	650.47	
	y ~ x + x2	p=0.10	0.06	652.61	
	y ~ x + x2 + x3	p=0.02	0.17	650.02	*
Infant nasal passage	y ~ x	p=0.36	<0.01	531.08	
	y ~ x + x2	p=0.52	<0.01	534.69	
	y ~ x + x2 + x3	p=0.23	0.02	535.58	
**Average rRNA copy number**					
	model	*P*-value	Adjusted R^2	BIC	Between-model Chi-square test significance
Volcanic ash	y ~ x	p=0.29	0.04	6.96	
	y ~ x + x2	p=0.04	0.56	0.70	*
	y ~ x + x2 + x3	* insufficient data points to fit model			

Glacier forefield	y ~ x	$p=0.25$	<0.01	17.01	
	y ~ x + x2	$p=0.39$	<0.01	20.34	
	y ~ x + x2 + x3	$p=0.20$	0.03	21.29	
Concrete seawall	y ~ x	$p<0.01$	0.09	9.53	
	y ~ x + x2	$p<0.01$	0.42	-14.80	***
	y ~ x + x2 + x3	$p<0.01$	0.46	-15.80	*
Water pipe	y ~ x	$p=0.74$	<0.01	22.43	
	y ~ x + x2	$p=0.03$	0.32	16.56	*
	y ~ x + x2 + x3	$p=0.07$	0.27	19.30	
Bumble bee gut	y ~ x	$p=0.21$	0.01	160.14	
	y ~ x + x2	$p=0.32$	<0.01	163.15	
	y ~ x + x2 + x3	$p=0.17$	0.05	163.88	
Teeth	y ~ x	$p<0.01$	0.19	35.02	
	y ~ x + x2	$p<0.01$	0.40	16.00	***
	y ~ x + x2 + x3	$p<0.01$	0.40	19.60	
Biofilm reactor	y ~ x	$p<0.01$	0.85	-127.66	
	y ~ x + x2	$p<0.01$	0.88	-134.88	***
	y ~ x + x2 + x3	$p<0.01$	0.91	-145.90	***
Infant nasal passage	y ~ x	$p=0.42$	<0.01	122.70	
	y ~ x + x2	$p=0.66$	<0.01	126.64	
	y ~ x + x2 + x3	$p=0.83$	<0.01	130.74	
**Average genome size**					
	model	*P*-value	Adjusted R^2	BIC	Between-model Chi-square test significance
Volcanic ash	y ~ x	$p<0.01$	0.88	238.71	ns
	y ~ x + x2	$p<0.01$	0.87	240.55	
	y ~ x + x2 + x3	* insufficient data points to fit model			
Glacier forefield	y ~ x	$p<0.01$	0.27	1513.72	
	y ~ x + x2	$p<0.01$	0.25	1517.51	
	y ~ x + x2 + x3	$p<0.01$	0.36	1512.77	**
Concrete seawall	y ~ x	$p<0.01$	0.27	1687.91	
	y ~ x + x2	$p<0.01$	0.32	1691.95	
	y ~ x + x2 + x3	$p<0.01$	0.34	1689.90	*
Water pipe	y ~ x	$p=0.97$	<0.01	509.96	
	y ~ x + x2	$p=0.26$	0.06	509.51	
	y ~ x + x2 + x3	$p=0.45$	<0.01	512.28	
	y ~ x	$p=0.21$	0.01	1332.23	

Bumble bee gut	y ~ x + x2	*p*=0.38	<0.01	1335.64	
	y ~ x + x2 + x3	*p*=0.36	<0.01	1337.98	
Teeth	y ~ x	*p*<0.01	0.59	1978.77	
	y ~ x + x2	*p*<0.01	0.62	1975.39	**
	y ~ x + x2 + x3	*p*<0.01	0.73	1955.34	***
Biofilm reactor	y ~ x	*p*=0.67	< 0.01	1264.64	
	y ~ x + x2	*p*<0.01	0.29	1251.14	**
	y ~ x + x2 + x3	*p*<0.01	0.71	1213.85	***
Infant nasal passage	y ~ x	*p*=0.04	0.05	1978.33	
	y ~ x + x2	*p*=0.02	0.09	1978.81	*
	y ~ x + x2 + x3	*p*<0.01	0.22	1972.23	**

Supplementary Table S5. The name, life-history classification, and group proportion (beta) of the top 10 KOs in each group identified by LDA.

(Separate Excel file)
